# Effect of neoadjuvant chemotherapy on overall survival of patients with T2-4aN0M0 bladder cancer: A systematic review and meta-analysis according to EAU COVID-19 recommendation

**DOI:** 10.1371/journal.pone.0267410

**Published:** 2022-04-21

**Authors:** Dong Hyuk Kang, Kang Su Cho, Young Joon Moon, Doo Yong Chung, Hae Do Jung, Joo Yong Lee

**Affiliations:** 1 Department of Urology, Inha University College of Medicine, Incheon, Korea; 2 Department of Urology, Gangnam Severance Hospital, Urological Science Institute, Yonsei University College of Medicine, Seoul, Korea; 3 Department of Urology, Severance Hospital, Urological Science Institute, Yonsei University College of Medicine, Seoul, Korea; 4 Department of Urology, Wonkwang University Sanbon Hospital, Wonkwang University College of Medicine, Gunpo, Korea; 5 Center of Evidence Based Medicine, Institute of Convergence Science, Yonsei University, Seoul, Korea; The University of Mississippi Medical Center, UNITED STATES

## Abstract

**Purpose:**

In the context of the COVID-19 outbreak, the European Association of Urology (EAU) guidelines Rapid Reaction Group provided recommendations to manage muscle invasive bladder cancer (MIBC) based on priority levels: neoadjuvant chemotherapy (NAC) should be avoided for patients with T2-3N0M0 MIBC. This meta-analysis aims to evaluate the efficacy of NAC compared with radical cystectomy (RC) alone in improving the overall survival (OS) of patients with T2-4aN0M0 MIBC.

**Materials and methods:**

A systematic review was performed according to the PRISMA guidelines. The PubMed/Medline, EMBASE, and Cochrane Library databases were searched. The primary outcome was OS of patients with T2-4aN0M0 MIBC, and the secondary outcome was OS of patients with only T2N0M0 MIBC.

**Results:**

Eight studies were included in this meta-analysis. Overall, the quality of all studies was relatively high, and little publication bias was demonstrated. The OS was significantly better in the NAC with RC group than in RC alone (HR, 0.79; 95% CI, 0.68–0.92; *p* = 0.002). A subgroup analysis was performed on only patients with T2N0M0 MIBC, and five studies were included. There was no difference in the OS between the NAC with RC and the RC alone groups (HR, 0.83; 95% CI, 0.69–1.01 *p* = 0.06).

**Conclusions:**

As recommended by the EAU guidelines Rapid Reaction Group, patients with T2N0M0 MIBC should strongly consider omitting NAC until the end of the COVID-19 pandemic. Whether to omit NAC in T3-4aN0M0 MIBC needs further discussion, and studies targeting only T2-3N0M0 MIBC are expected to proceed further.

## Introduction

With the spread of coronavirus disease 2019 (COVID-19), the world is experiencing an unprecedented time in history, and the medical field is undergoing major changes. Many medical and surgical procedures have been postponed or omitted because of the COVID-19 pandemic [1, 2]. The basis for this approach is to reduce the risk of COVID-19 transmission, increase bed availability for patients with COVID-19 in wards and intensive care units, reduce the workload of healthcare providers except for COVID-19 cases, and limit aerosol generation procedures. For urological diseases, urologists should also manage patients with COVID-19 and urological diseases in a balanced manner. Considering the COVID-19 pandemic, the European Association of Urology (EAU) guidelines Rapid Reaction Group presents a response policy for diagnosis, treatment, and follow-up according to the priority of urologic diseases, including urological cancer [3]. The guidelines were also presented for bladder cancer (BCa), and they recommend that neoadjuvant chemotherapy (NAC) should be omitted in patients with T2-3 focal N0M0 muscle-invasive bladder cancer (MIBC) during the COVID-19 pandemic.

Of all urogenital cancers, it is difficult to make timely decisions about BCa. Patients with BCa are generally older, more susceptible to COVID-19, and often have several comorbidities. In particular, MIBC often requires both chemotherapy and radical cystectomy (RC), which can cause morbidity and mortality. Cisplatin-based NAC followed by RC is the standard of care and recommended treatment for clinical stage II and IIIA BCa [4, 5]. Chemotherapy can be performed before or after surgery [6], but there are several criteria for NAC. The first is to treat micrometastatic disease at the time of diagnosis when the burden of disease is the lowest [7]. In addition, patients are more likely to tolerate preoperative chemotherapy than postoperative chemotherapy [6].

Several meta-analyses on the efficacy of NAC for MIBC reported that NAC is effective [8, 9]. However, one study reported that there was no difference in NAC compared with cystectomy and/or radiotherapy alone [10]. Disagreements in these studies require a more comprehensive analysis to encourage the use of NAC in MIBC treatment. We intend to perform an updated meta-analysis by extracting studies targeting patients with T4a or lower without lymph node metastasis among MIBC and adding recently published papers. Furthermore, through this meta-analysis, we would like to examine the evidence for the omission of NAC in patients with T2-3N0M0 MIBC as recommended by the EAU guidelines Rapid Reaction Group in more detail.

## Materials and methods

### Literature search and study selection

A comprehensive literature search was conducted until October 31, 2021, using PubMed Central, Cochrane Central Controlled Register of Trials (CENTRAL), and Embase. We used search terms such as “muscle-invasive bladder cancer,” “neoadjuvant chemotherapy,” “cystectomy,” and “overall survival” and relevant variants. Titles and abstracts were screened for relevance, followed by full-text screening. All duplicate articles were excluded, and related articles were finally searched from the reference list of the retrieved articles. This meta-analysis was registered on the PROSPERO website (number: CRD42021299238).

### Inclusion and exclusion criteria

This systematic review and meta-analysis followed the participants, interventions, comparators, outcomes, and study design (PICOS) approach and Preferred Reporting Items for Systematic Reviews and Meta-Analyses (PRISMA) guidelines. The PICOS model for this meta-analysis consisted of Participants (patients with T2-4aN0M0 MIBC), Intervention (T2-4aN0M0 MIBC patients who underwent neoadjuvant chemotherapy), Comparison (T2-4aN0M0 MIBC patients who underwent RC alone), Outcome (comparison of overall survival (OS)), and Study type (randomized clinical trials, prospective, and retrospective studies). The following inclusion criteria were adopted: patients with MIBC proven by histological examination; two-arm studies that compared NAC with RC and RC alone; no lymph node and distant metastases; and studies with OS outcomes. The exclusion criteria were: studies that compared different NAC regimens without RC, single-arm studies, case reports, reviews, commentaries, animal studies, and non-English written studies.

### Data extraction and quality assessment

All the identified articles were independently screened by two reviewers (DHK and HDJ), and two other reviewers (DYC and YJM) independently analyzed all the details of each article to confirm that they met the inclusion criteria. Any discrepancy between the two reviewers was resolved through discussion until agreed, or via third-party adjudication performed by another reviewer (JYL). Once the final group of articles was agreed upon, two researchers independently examined the quality. The quality of each study was estimated using the Cochrane risk-of-bias tool for randomized control trials (RCTs) [11] and the Newcastle–Ottawa Scale (NOS) for non-randomized controlled trials (www.ohri.ca/programs/clinical_epidemiology/oxford.asp) [12].

## Data synthesis and analysis

The primary outcome measures were OS of patients with T2-4aN0M0 MIBC, and secondary outcome measures were OS of patients with only T2N0M0 MIBC. Meta-analysis was conducted using R (R version 4.1.3, R Foundation for Statistical Computing, Vienna, Austria; http://www.r-project.org) and its meta and metasens packages. The effect measures of the outcomes of interest were the hazard ratios (HRs) with 95% confidence interval (CI), which was obtained by extracting the proportion of patients with the outcome events. Pooled incidences were calculated using a fixed-effects or random-effects model according to the heterogeneity of the included studies. The Cochrane Q and I^2^ statistics were used to assess the statistical heterogeneity. If no significant statistical inconsistency was observed (I^2^ < 25%), the summary estimate was calculated using the fixed-effects model. When heterogeneity was observed, the summary estimate was calculated using the random-effects model.

### Ethics statement

The data and results used in this paper are all from published studies, and there is no ethical issue, so the approval of the ethics committee is not required.

## Results

### Study characteristics

**Fig 1:**
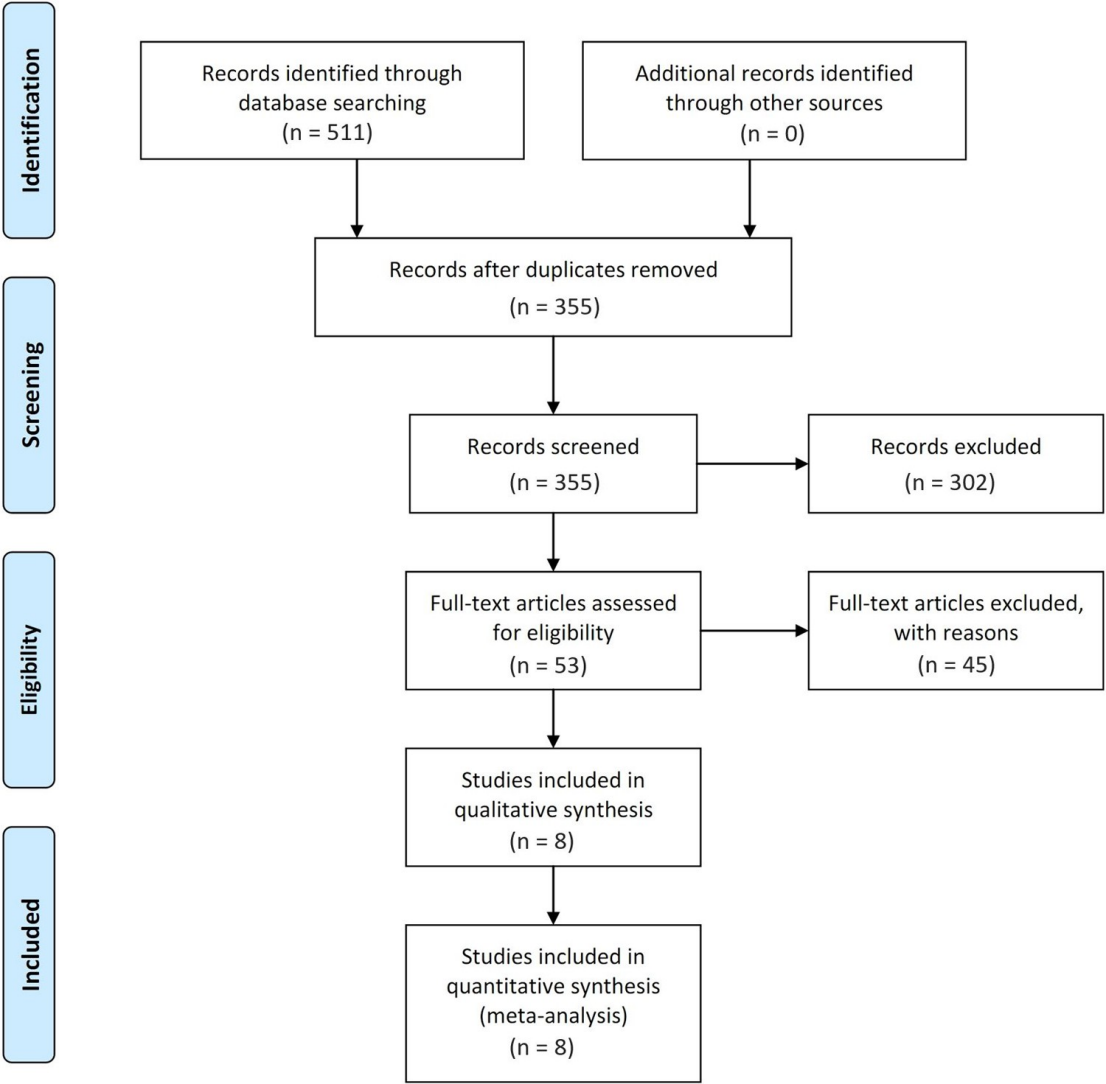
Search strategy for a systematic review and meta-analysis.

The database searches identified 355 articles that were included in this meta-analysis. According to the inclusion and exclusion criteria, 302 articles were excluded after a brief review of the titles and abstracts of the articles. This left 53 studies that evaluated the OS. After reviewing the full-text articles of these studies, 45 were excluded due to irrelevant results. Fig 1 shows the results of the comprehensive search, selection process, and number of excluded studies along with the relevant evidence. The search finally identified eight studies [13–20]. Two studies used methotrexate/vinblastine/doxorubicin/cisplatin (MVAC), one study used gemcitabine/cisplatin (GC), one study used cisplatin or carboplatin-based regimen, and one study used cisplatin-based NAC, remaining three studies did not mention the chemotherapy regimen. The characteristics of the included studies are presented in Table 1.

**Table 1:** Characteristics of included studies.

Study	Year	Type of Study	Stage	No of patients			Age (years)		Sex (Male:Female)		5-year survival rate (%)		Median OS (months)		NAC Regimen
				Total	NAC+RC	RC alone	NAC+RC	RC alone	NAC+RC	RC alone	NAC+RC	RC alone	NAC+RC	RC alone	
Grossman et al. [13]	2003	RCT	T2-4a	307	153	154	63	63	127:26	124:30	NA	NA	77	46	MVAC
Kitamura et al. [14]	2014	RCT	T2-4a	130	64	66	63	63	57:7	60:6	72.3	62.4	102	82	MVAC
Osman et al. [15]	2014	Clinical trial	T2-4a	60	30	30	48.9	52.3	28:2	29:1	60 (3-year)	50 (3-year)	36+	32.5	GC
Lane et al. [16]	2018	Retrospective	T2	1886 (Before PW)	381 (Before PW)	1505 (Before PW)	NA	NA	292:89	1140:365	NA	NA	NA	NA	Cisplatin or Carboplatin based
Mazzone et al. [17]	2018	Retrospective	T2	3978 (Before IPTW)	1519 (Before IPTW)	2459 (Before IPTW)	66	69	1193:326	1895:564	54.6 (10-year)	57.9 (10-year)	NA	NA	NA
Hermans et al. (1) [18]	2019	Retrospective	T2	4355	191	4164	NA	NA	NA	NA	51	57	NA	NA	NA
Hermans et al. (2) [18]	2019	Retrospective	T3-4a	967	133	834	NA	NA	NA	NA	54	36	NA	NA	NA
Hermas et al. (1) + (2) [18]	2019	Retrospective	T2-4a	5322	324	4998	63	67	238:	3820:	NA	NA	NA	NA	NA
Russell et al. [19]	2019	Retrospective	T2	370	183	187	NA	NA	NA	NA	NA	NA	NA	NA	NA
Soria et al. [20]	2021	Retrospective	T2	619	316	303	64	69	266:50	240:63	73 (2yr)	60 (2yr)	NA	NA	usually Cisplatin based

OS: overall survival, NAC: neoadjuvant chemotherapy, RC: radical cystectomy, RCT: randomized controlled trial, MVAC: methotrexate/vinblastine/doxorubicin/cisplatin, GC: gemcitabine/cisplatin, PW: propensity-weighting, IPTW: inverse probability of treatment-weighting

### Primary endpoint: Outcomes of T2-4aN0M0 MIBC patients

#### Heterogeneity assessment

**Fig 2:**
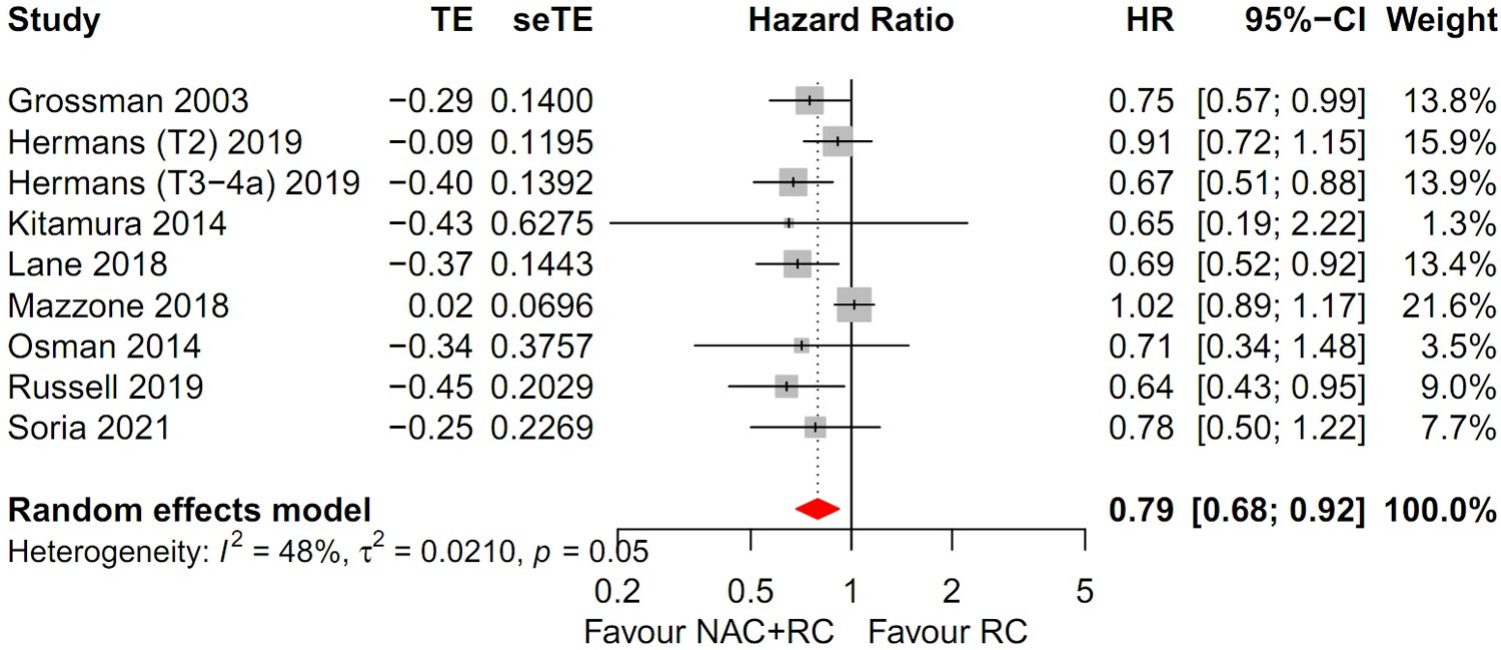
Forest plots for patients with T2-4aN0M0 MIBC. The overall survival was significantly better in the NAC with RC group than in the RC alone group (HR, 0.79; 95% CI, 0.68–0.92; *p* = 0.002).

**Fig 3:**
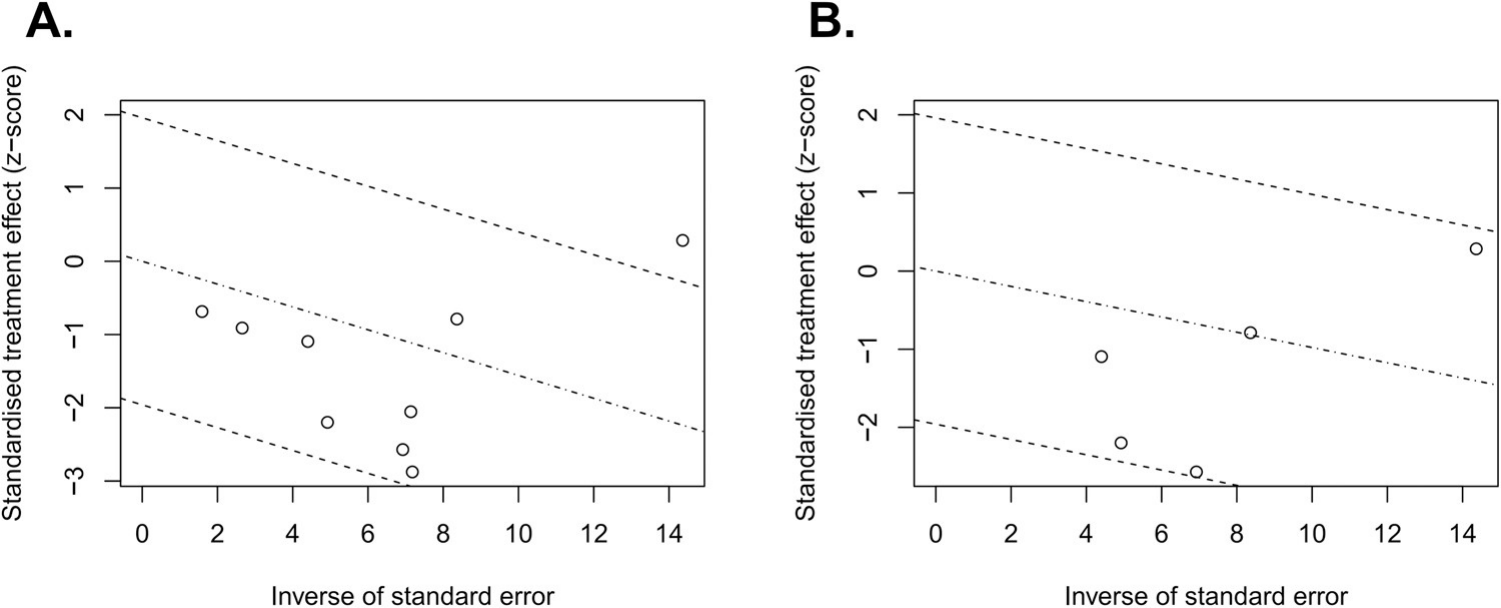
Radial plots of the overall survival of patients with T2-4aN0M0 MIBC (A) and patients with T2N0M0 MIBC (B). After selection of the effect models, little heterogeneity was observed in the radial plots.

**Fig 4:**
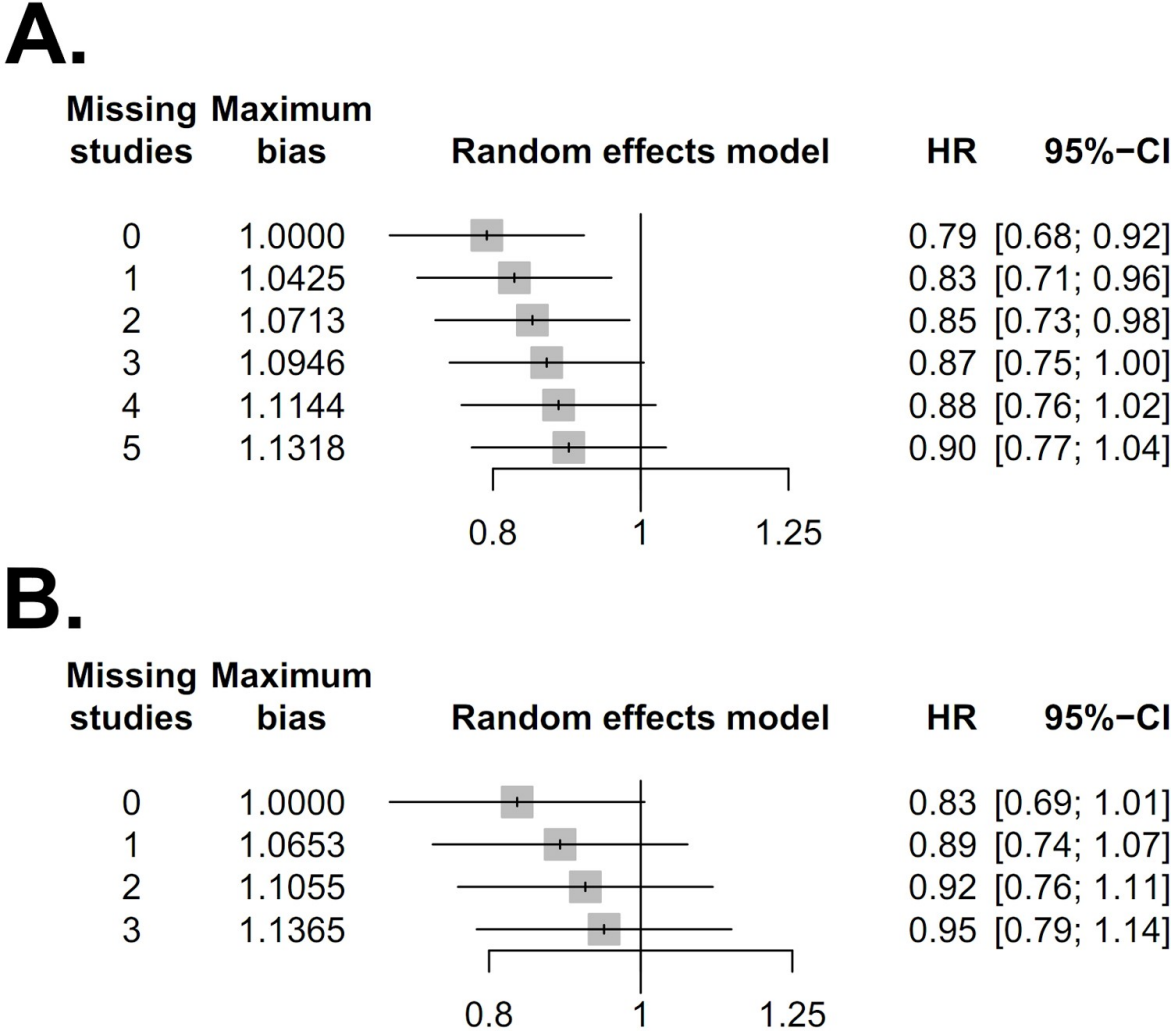
Sensitivity analysis for the outcome reporting bias (ORB) of patients with T2-4aN0M0 MIBC (A) and patients with T2N0M0 MIBC(B). The sensitivity was considered robust in the sensitivity analysis for ORB.

Eight trials accounting for a total of 12,672 assessable patients were included in the analysis. Forest plots of patients with T2-4aN0M0 MIBC are shown in Fig 2. There was high heterogeneity (I^2^ = 48%, *p* = 0.05), hence, a random-effects model was used. After selection of the effect models, little heterogeneity was observed in the radial plots (Fig 3A). We conducted sensitivity analysis for outcome reporting bias (ORB) to examine the degree of heterogeneity (Fig 4A). The sensitivity of this meta-analysis was considered robust, as the results on OS were not affected until up to two studies were excluded.

#### Overall survival

The OS was significantly higher in the NAC with RC group than in RC alone group (HR, 0.79; 95% CI, 0.68–0.92; *p* = 0.002) (Fig 2).

### Secondary endpoint: Outcomes of T2N0M0 MIBC patients

#### Heterogeneity assessment

**Fig 5:**
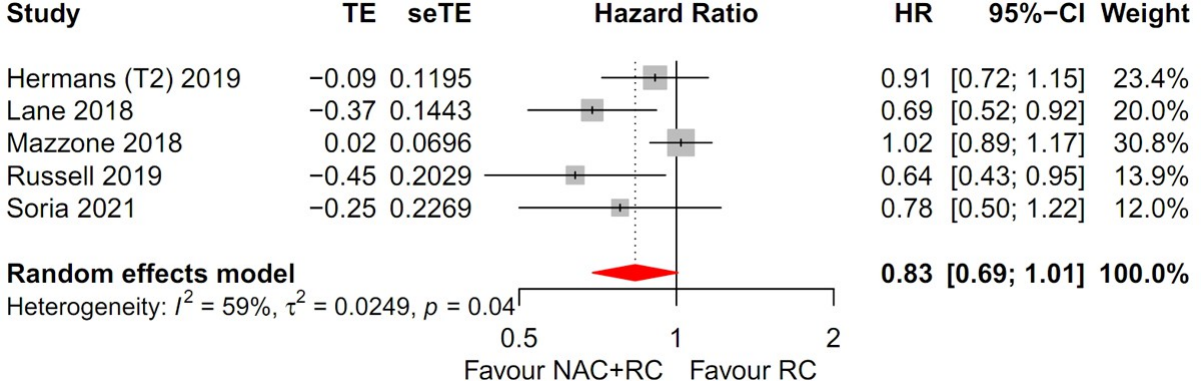
Forest plots for patients with T2N0M0 MIBC. There was no difference in overall survival between the NAC with RC group and RC alone group (HR, 0.83; 95% CI, 0.69–1.01; *p* = 0.06).

Five trials accounting for a total of 11,208 assessable patients were included in the analysis. Forest plots of patients with T2N0M0 MIBC are shown in Fig 5. There was high heterogeneity (I^2^ = 59%, *p* = 0.04), thus a random-effects model was used. After selection of the effect models, little heterogeneity was observed in the radial plots (Fig 3B). We conducted sensitivity analysis for ORB to examine the degree of heterogeneity (Fig 4B). The sensitivity of this meta-analysis was considered robust, as excluding the study did not affect the results on OS.

#### Overall survival

There was no difference in OS between the NAC with RC group and the RC alone group (HR, 0.83; 95% CI, 0.69–1.01; *p* = 0.06).

### Quality assessment

The results of the quality assessment based on the Cochrane risk-of -bias tool of the three included RCTs are shown in Table 2A. Cancer treatment consisting of NAC and surgery informed both clinicians and patients of the treatment and provided written consent to all patients. Therefore, the high risk of bias in allocation concealment and blind processes could not be avoided. The results of the quality assessment using the NOS for the included nonrandomized studies are shown in Table 2B. All five studies received a score of 6 points (indicating high quality).

**Table 2:** Results of quality assessment by the Cochrane risk-of- bias tool (A) and NOS (B).

A. Quality assessment of a randomized controlled trial									
Author(s) (Year)	Random Sequence Generation (Selection Bias)		Allocation Concealment (Selection Bias)	Blinding of Participants and Personnel (Performance Bias)	Blinding of Outcome Assessment (Detection Bias)		Incomplete Outcome Data Addressed (Attrition Bias)	Selective Reporting (Reporting Bias)	Other Bias
Grossman et al. (2003) [13]	Low risk		High risk	High risk	High risk		Low risk	Low risk	Unclear
Kitamura et al. (2014) [14]	Low risk		High risk		High risk	High risk		Low risk	Low risk	Unclear
Osman et al. (2014) [15]	Unclear		High risk	High risk	High risk		Low risk	Low risk	Unclear
B. Quality assessment of nonrandomized studies									
Author(s) (Year)	Selection (4)				Comparability (2)	Exposure (3)			Total score
	Adequate Definition of Cases	Representativeness of Cases	Selection of Controls	Definition of Controls	Control for Important Factor or Additional Factor	Ascertainment of Exposure	Same Method of Ascertainment for Cases and Controls	Non-Response Rate	
Lane et al. (2018) [16]	1	1	0	0	2	1	1	0	6
Mazzone et al. (2018) [17]	1	1	0	0	2	1	1	0	6
Hermans et al. (2019) [18]	1	1	0	0	2	1	1	0	6
Russell et al. (2019)	1	1	0	0	2	1	1	0	6
Soria et al. (2021)	1	1	0	0	2	1	1	0	6

NOS: Newcastle–Ottawa Scale

### Publication bias

**Fig 6:**
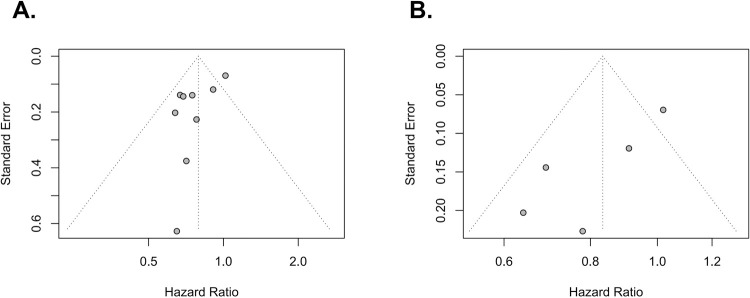
Funnel plots for patients with T2-4aN0M0 MIBC (A) and patients with T2N0M0 MIBC (B). There was little publication bias in funnel plots.

Funnel plots from these meta-analyses are shown in Fig 6. Little publication bias was observed in the funnel plots.

## Discussion

The results of this study confirmed that NAC with RC was more helpful in the survival of patients than RC alone in patients with T2-4aN0M0 MIBC, and that there was no difference in survival between the two groups of RC alone and NAC with RC in patients with T2N0M0 MIBC. The main purpose of this study was to corroborate the evidence presented by the EUA guidelines Rapid Reaction Group on omitting NAC in patients with T2-3N0M0 MIBC during the COVID-19 pandemic [3] through a met-analysis. The authors identified solid evidence in patients with T2N0M0 MIBC. Few studies have targeted patients with only T2-3N0M0, thus it was difficult to substantiate the guidelines for T3N0M0. However, in patients with T2-4aN0M0 MIBC, it was statistically confirmed that NAC with RC helped the patients’ survival compared with RC alone, but compared with T2N0M0, HR did not differ much (HR 0.79 vs 0.83). Therefore, it can be inferred from this study that there is a sufficient possibility that there is no statistical difference if only T2-3N0M0 is targeted.

COVID-19 is an acute respiratory infectious disease caused by SARS-CoV-2, a novel strain of coronavirus that was first reported in November 2019 [21]. The COVID-19 pandemic has had major effects on individuals and healthcare systems. Limited healthcare resources, as well as those infected with SARS-CoV-2, have contributed to the spread of the virus. Thus resulting in reduced capacity and rapid depletion of health systems and hospitals [22]. To prevent the spread of the virus, hospital visits should be limited by delaying surgery or procedures, or by omitting low-priority treatments [23]. However, medical delays or omissions can increase the morbidity and risk of death from treatable and preventable diseases. Since there are few international medical emergency situations historically, it is difficult to judge the pros and cons of delaying or avoiding treatment for any disease. Therefore, preparing individual guidelines for each disease is very important in the current COVID-19 pandemic.

There have been many changes in the field of urology due to COVID-19. Many urologists are making great efforts not only for the management of COVID-19, but also for the management of urologic diseases in the current situation. In response to the COVID-19 pandemic, the EAU Guidelines Office has been working with the Executive Committee, the Section offices, and others to set up a “Rapid Reaction Group” [3]. The protocol was divided into four large categories to provide recommendations: diagnosis, surgical treatment and medical therapy, follow-up/telemedicine, and emergency. The panel provides a table with recommendations according to priority level. Four color-separated risk stratification tools were created to help apply recommendations and are as follows: low priority, clinical harm very unlikely if postponed for 6 months (green); intermediate priority: clinical harm possible, but unlikely, if postponed for 3–4 months (yellow); high priority: clinical harm very likely if postponed for >6 weeks (red); emergency: life-threatening situation–cannot be postponed for >24 h (black).

All urological cancers, including BCa, were evaluated by the EAU guidelines Rapid Reaction Group. Among urological cancers, the managements for BCa can be the most diverse treatments. Depending on the stage or grade, there are a wide variety of treatments such as active surveillance, transurethral resection of bladder tumor, intravesical bacillus Calmette-Guérin instillation, RC, chemotherapy, and radiotherapy [24]. During the COVID-19 pandemic, the EAU Guidelines Office presented a diverse set of guidelines depending on the status of BCa, including omitting NAC for patients with T2-3 focal N0M0 MIBC (green color by the EAU guidelines Rapid Reaction Group). While these guidelines have been provided by highly-experienced and respected board and panel members, scientific evidence may be lacking as the guidelines were developed in a short period of time. Thus, we concluded that a meta-analysis of NAC with RC vs. RC alone in patients with MIBC was necessary to support the rationale for the guideline. In patients with stage II and IIIA BCa, post-NAC, RC may be the standard treatment, or RC alone may also be an option (especially for those who are not eligible for cisplatin-based chemotherapy) [4, 5]. The EAU Guidelines Office recommends omitting NAC in patients with stage II and stage IIA BCa during the COVID-19 pandemic. We paid attention to this recommendation and concluded that it is very important to confirm the extent to which NAC actually helps survival in patients with T2-3N0M0 through meta-analysis.

Prior to this study, several meta-analyses have analyzed the effect of NAC with RC on survival in patients with MIBC compared to RC alone. A meta-analysis based on 11 trials, 3005 patients; comprising 98% of all patients from known eligible RCTs, reported that the cisplatin-based combination NAC resulted in an absolute OS benefit of 5% (HR 0.86 [0.77–0.95]) at 5 years [8]. Recently, Hamid et al. [9] conducted a meta-analysis of 17 studies with 13,391 patients and reported that the results showed similar HR (HR 0.82 [0.71–0.95]) to the aforementioned study and that NAC improved OS. However, another meta-analysis reported that there was no difference in NAC with RC compared with RC and/or radiotherapy (HR 0.92 [0.84–1.00]) [10]. The results of the studies have some discrepancies due to minor differences in patient groups and treatments in each meta-analysis, including the present study. However, the inconsistencies of these study results indicate that a comprehensive analysis is necessary to determine the effect of NAC on the treatment of MIBC, and we suggested that the results of present study can help reach a consensus to some extent. In addition, our study has the advantage of providing more specific information because it limited patients up to T4a among patients without lymph node metastasis. In particular, in the current crisis of medical systems during COVID-19, it is important to efficiently use the limited medical resources through a balanced approach between the management of infectious diseases and the treatment of cancer. Our study can be helpful to establish the treatment guidelines for this global emergency.

In this study, NAC had an OS advantage in T2-4aN0M0 patients, but no OS benefit in T2N0M0 patients. The role of preoperative NAC in MIBC is well known. Patients can tolerate chemotherapy better, and it also makes surgery easier by reducing the tumor burden during surgery [25]. In addition, if there is micrometastasis, it can be effectively treated [7]. These points act as an advantage; and in this study, it was considered that the OS in patients with T2-4aN0M0 was also beneficial. On the other hand, several reasons can be assumed as to why the OS did not show any benefit when only T2N0M0 patients were targeted. First, since T2 BCa can be considered as an organ-confined status, reducing the tumor burden may not have a significant effect [26]. Also, since the presence of micrometastases is relatively lower, NAC may not be effective. In addition, in patients in whom NAC is ineffective, delay in surgery due to NAC may actually cause disease progression [27]. Appropriate additional research is needed in this regard.

This study has some limitations. First, only T2-3N0M0 as suggested by the EAU guidelines Rapid Reaction Group was not separately classified. In most clinical studies, it was difficult to separate patients with T2-3N0M0 from patients with lymph nodes positive, T2-4, T2-4a, T3-4, or T2. However, T4a is a case of invasion of the prostate, seminal vesicles, uterus, or vagina, which can still be defined as a surgically resectable status. T4b is a case in which the pelvic wall or abdominal wall is invaded, and complete surgical removal is considered impossible. Therefore, T4a was included in this study and T4b was excluded, and the patient group was reduced as close to the guidelines presented by the EAU guidelines Rapid Reaction Group as much as possible. In addition, subgroup analysis was performed on only patients with T2N0M0, and the evidence of the guidelines was clearly corroborated for T2N0M0. Additionally, we did not investigate the side effects or quality of life associated with NAC. In a prospective study, NAC was found to be associated with a 30%–40% rate of grade 3–4 toxicity [28]. In addition, most patients are elderly and often have comorbidities, so it is appropriate to consider the side effects and quality of life associated with NAC as well as other oncologic outcomes. Moreover, detailed analysis according to the regimen or cycle of NAC was not performed. Incomplete chemotherapy has been recently associated with pathological progression during NAC and an inferior pathological response after RC [29, 30]. Therefore, when evaluating patient outcomes, it is necessary to carefully consider the regimen and cycle of NAC. Hence, additional studies are needed in the near future. Lastly, the studies included in this meta-analysis showed a high degree of heterogeneity. This was probably due to the large difference in weight between the studies, indicating high heterogeneity. To overcome this issue, we analyzed heterogeneity through radial plots after applying the random-effects model, and observed almost no heterogeneity. In addition, it was analyzed to show robust sensitivity through sensitivity analysis for ORB.

Nevertheless, this study is the first analysis to substantiate the evidence presented by the EAU guidelines Rapid Reaction Group to omit NAC from the T2-3 focal N0M0 MIBC during the COVID-19 pandemic, and its impact can be unique. Unfortunately, the T2-3N0M0 group was not analyzed due to the limited number of studies, but strong evidence was found in the T2N0M0 group. In addition, we tried to derive the results by narrowing T stages down to T4a or less, and we think that this study can positively support the COVID-19 EAU guideline when the results of this study are comprehensively considered. In the future, several studies on patients with T2-3N0M0 MIBC should be published, and studies examining side effects, quality of life, and oncological outcomes will be needed.

## Conclusion

As recommended by the EAU guidelines Rapid Reaction Group, patients with T2N0M0 MIBC should strongly consider omitting NAC until the end of the COVID-19 pandemic. Whether to omit NAC in patients with T3-4aN0M0 MIBC needs further discussion, and studies targeting only T2-3N0M0 MIBC are expected to proceed further.

## Supporting information

S1 Checklist PRISMA checklist. (DOCX)
